# Adjunctive hyperbaric oxygen therapy for post-stroke cognitive impairment: a systematic review and meta-analysis of randomized controlled trials

**DOI:** 10.3389/fneur.2026.1982226

**Published:** 2026-10-13

**Authors:** Xiaosai Hu, Shurong Zhao, Dandan Zhang, Xianguo Meng

**Affiliations:** 1 School of Sports Medicine and Rehabilitation, Shandong First Medical University (Shandong Academy of Medical Sciences), Taian, China; 2 Shandong First Medical University (Shandong Academy of Medical Sciences), Jinan, China; 3 Department of Rehabilitation Medicine, The First Affiliated Hospital of Shandong First Medical University & Shandong Provincial Qianfoshan Hospital, Jinan, China

**Keywords:** cognitive function, hyperbaric oxygen therapy, meta-analysis, post-stroke cognitive impairment, randomized controlled trials

## Abstract

**Objective:**

To evaluate the effects of adjunctive hyperbaric oxygen therapy (HBOT) on cognitive, neurological, and functional outcomes in patients with post-stroke cognitive impairment (PSCI), and explore potential sources of heterogeneity.

**Methods:**

Eight databases were searched from inception to July 15, 2026. Randomized controlled trials (RCTs) evaluating adjunctive HBOT in patients with PSCI were included. The primary outcome was the Montreal Cognitive Assessment (MoCA); secondary outcomes included the Mini-Mental State Examination (MMSE), National Institutes of Health Stroke Scale (NIHSS), Modified Barthel Index (MBI), and Barthel Index (BI). Random-effects meta-analyses, prespecified subgroup analyses, and meta-regression were performed. Sensitivity analyses, funnel plots, Egger’s tests, and GRADE assessments were conducted.

**Results:**

Thirty-five RCTs involving 3,036 participants, all conducted in China, were included. Adjunctive HBOT was associated with higher MoCA scores (MD = 2.90, 95% CI: 2.47–3.33; *p* < 0.00001; *I*^2^ = 84%) and MMSE scores (MD = 2.68, 95% CI: 2.20–3.16; *p* < 0.00001; *I*^2^ = 83%). Improvements were also observed in NIHSS (MD = −2.61, 95% CI: −3.34 to −1.89; *p* < 0.00001; *I*^2^ = 93%), MBI (MD = 6.64, 95% CI: 3.76–9.52; *p* < 0.00001; *I*^2^ = 82%), and BI (MD = 8.77, 95% CI: 4.72–12.82;*p* < 0.00001; *I*^2^ = 82%). No statistically significant subgroup differences were observed for MoCA (*p* = 0.10) or MMSE (*p* = 0.43). Higher baseline MoCA scores tended to be associated with smaller treatment effects in meta-regression (*β* = −0.1411, *p* = 0.0637; *R*^2^ = 15.42%). Substantial heterogeneity remained largely unexplained. Evidence certainty was low for MoCA, MMSE, and MBI and very low for NIHSS and BI.

**Conclusion:**

Adjunctive HBOT may provide cognitive and functional benefits in patients with PSCI. However, substantial unexplained heterogeneity, low-to-very-low certainty of evidence, and the exclusively Chinese evidence base warrant cautious interpretation and limit generalizability. Further well-designed RCTs involving diverse populations are needed.

**Systematic review registration:**

https://www.crd.york.ac.uk/PROSPERO/view/CRD420261473579, PROSPERO, CRD420261473579.

## 1 Introduction

Stroke is a leading cause of death and disability worldwide and imposes a substantial global health burden (1). According to the World Stroke Organization Global Stroke Fact Sheet 2025, nearly 12 million new strokes occur annually, with approximately 93.8 million stroke survivors worldwide (2). With population aging and persistent stroke risk factors, the long-term functional and socioeconomic consequences of stroke have received increasing attention (3, 4).

Post-stroke cognitive impairment (PSCI) is a common complication of stroke, affecting multiple cognitive domains, including attention, executive function, memory, and visuospatial abilities, and is associated with reduced functional independence and quality of life (5). Recent systematic reviews and meta-analyses have reported a high prevalence of persistent cognitive impairment after stroke, highlighting PSCI as an important target in long-term stroke rehabilitation and management (6).

Current management of PSCI includes pharmacological therapy, cognitive training, physical rehabilitation, and neuromodulation (7–9). Although these interventions may improve cognitive and functional outcomes, their efficacy varies, and effective therapies targeting the underlying mechanisms of post-stroke neural injury remain limited (10). Therefore, identifying safe, feasible, and potentially beneficial therapeutic approaches is of considerable importance for promoting cognitive and functional recovery in patients with PSCI.

Hyperbaric oxygen therapy (HBOT) involves breathing high-concentration oxygen under pressures greater than atmospheric pressure, thereby increasing tissue oxygen availability. HBOT has attracted increasing attention in neurological rehabilitation and may exert neuroprotective effects by improving tissue oxygenation and modulating oxidative stress, apoptosis, mitochondrial function, and neurovascular remodeling (11–14). These neuroprotective effects may also support cognitive recovery after stroke by helping preserve neuronal function in injured brain regions, although the mechanisms underlying cognitive improvement following HBOT remain incompletely understood (13, 15). Previous preclinical and clinical studies have suggested that HBOT may improve neurological deficits and cognitive dysfunction following stroke (14, 16, 17).

However, evidence specifically addressing HBOT for PSCI remains limited. Previous systematic reviews of HBOT have primarily focused on acute ischemic stroke or broader neurological and functional outcomes, while PSCI reviews have mainly examined cognitive training, neuromodulation, and other non-pharmacological interventions (18–20). Moreover, existing HBOT trials vary in patient characteristics and treatment protocols, and the effects of HBOT on PSCI remain uncertain.

We therefore conducted a systematic review and meta-analysis of RCTs to evaluate the efficacy and safety of adjunctive HBOT in patients with PSCI and explore potential sources of between-study heterogeneity.

## 2 Methods

This systematic review was registered prospectively with PROSPERO (registration number: CRD420261473579).

### 2.1 Search strategy

A systematic literature search was conducted in eight electronic databases: PubMed, Embase, Cochrane Library, Web of Science, Scopus, China National Knowledge Infrastructure (CNKI), Wanfang Data, and VIP. The databases were searched from inception to July 15, 2026, for potentially eligible studies. The search strategy was developed around three core concepts: “stroke,” “post-stroke cognitive impairment,” and “hyperbaric oxygen therapy,” using a combination of controlled vocabulary and free-text terms. Chinese search terms included “脑卒中,” “脑梗死,” “卒中后认知障碍,” “认知,” and “高压氧,” together with their relevant synonyms. English search terms included “stroke,” “cerebral infarction,” “post-stroke cognitive impairment,” “cognitive,” and “hyperbaric oxygen therapy,” along with their related synonyms. Search fields and controlled vocabulary were adapted according to the characteristics of each database. In addition, the reference lists of included studies and relevant systematic reviews were manually screened to identify potentially eligible studies and minimize the possibility of missing relevant evidence. The complete search strategies and specific search strings for each database are provided in the Supplementary materials.

### 2.2 Eligibility criteria

*Inclusion criteria*: (1) patients with a clinical diagnosis of stroke and concomitant cognitive impairment; (2) participants in the intervention group received HBOT; (3) participants in the control group received the same treatment as the intervention group but without HBOT; (4) the primary outcome was the Montreal Cognitive Assessment (MoCA) score, while secondary outcomes included the Mini-Mental State Examination (MMSE), National Institutes of Health Stroke Scale (NIHSS), Modified Barthel Index (MBI), Barthel Index (BI), and other cognitive or functional outcomes; and (5) the study design was a randomized controlled trial (RCT).

*Exclusion criteria*: (1) non-randomized controlled trials; (2) duplicate publications or multiple reports from the same study population. When multiple reports from the same study were identified, the report with the most complete information or the greatest relevance to the outcomes of interest was retained; (3) conference abstracts, reviews, and other non-original research reports; (4) studies for which the full text could not be obtained, or for which key study data remained unavailable after necessary verification and contact with the authors; (5) studies were excluded only when they did not meet the prespecified eligibility criteria; and (6) studies published in languages other than Chinese or English; (7) No geographical restrictions were imposed on study eligibility. Although no geographical restrictions were imposed, all eligible studies identified in this review were conducted in China.

### 2.3 Study selection and data extraction

Two investigators (HXS and ZSR) independently screened the literature and extracted data, with all extracted information cross-checked for consistency. Disagreements were resolved through discussion; when consensus could not be reached, a third investigator (MXG) made the final decision. A prespecified data extraction form was used to collect relevant information, including study characteristics (study name, sample size, sex distribution, age, etc.), intervention characteristics and treatment parameters (treatment pressure and number of treatment sessions, etc.), and baseline and post-intervention data for the outcome measures. For studies with missing or unclear information, the full text and Supplementary materials were first reviewed for clarification. When necessary, the corresponding authors were contacted to obtain or verify the relevant data.

### 2.4 Risk of bias assessment

The risk of bias in the included RCTs was assessed using the Cochrane Risk of Bias 2 (RoB 2) tool. The tool covers five domains: bias arising from the randomization process, bias due to deviations from intended interventions, bias due to missing outcome data, bias in measurement of the outcome, and bias in selection of the reported result. Each domain was assessed using the corresponding signaling questions, and the risk of bias was classified as low risk, some concerns, or high risk according to the tool’s decision rules. An overall risk-of-bias judgment was then made based on the domain-level assessments.

### 2.5 Statistical analysis

Statistical analyses were performed using Review Manager (RevMan) version 5.4.1 and R software version 4.4.1, with the meta package used for all statistical analyses conducted in R. Continuous outcomes were summarized as mean differences (MDs) with 95% confidence intervals (CIs). Given the potential clinical differences among studies in terms of participant characteristics, intervention protocols, and treatment procedures, random-effects meta-analyses were performed, with between-study variance (τ^2^) estimated using the DerSimonian–Laird (DL) method. Statistical heterogeneity across studies was assessed using Cochran’s *Q* test and the *I*^2^ statistic.

To explore potential sources of between-study heterogeneity, prespecified subgroup analyses were conducted according to the type of conventional treatment received by both the intervention and control groups. Studies were categorized into medication treatment, physical rehabilitation, cognitive rehabilitation, acupuncture, and repetitive transcranial magnetic stimulation (rTMS) subgroups. Differences in effect estimates between subgroups were assessed using tests for subgroup differences. Given the limited stability of effect estimates in subgroups with few studies, formal comparisons of subgroup effects were not performed for subgroups containing three or fewer studies. In addition, random-effects meta-regression analyses were performed in R to investigate associations between study characteristics and effect estimates. Prespecified potential covariates included the total number of HBOT sessions (Sessions), baseline MoCA score at study enrollment (Baseline_MoCA), and HBOT treatment pressure (Pressure). Sessions, Baseline_MoCA, and Pressure were treated as continuous variables.

Leave-one-out sensitivity analyses were performed by sequentially removing each included study and repeating the meta-analysis to assess the influence of individual studies on the pooled estimates and between-study heterogeneity, thereby evaluating the robustness of the primary findings. When at least 10 studies were available for a given outcome, funnel plots were used to assess potential small-study effects, and Egger’s linear regression test was performed for quantitative assessment. Funnel plots and Egger’s tests were not performed when fewer than 10 studies were available. A two-sided *p* < 0.05 was considered statistically significant, except for Egger’s test, for which *p* < 0.05 was considered indicative of potential small-study effects.

### 2.6 Certainty of evidence

The certainty of evidence for the primary and important secondary outcomes was assessed using the GRADEpro GDT online tool. According to the GRADE framework, evidence from randomized controlled trials initially starts at high certainty and is assessed across five domains: risk of bias, inconsistency, indirectness, imprecision, and publication bias. The certainty of evidence was downgraded when relevant limitations were identified, according to their severity. The overall certainty of evidence was classified into four levels: high, moderate, low, and very low.

## 3 Results

### 3.1 Search results

A total of 1,041 records were identified from eight databases: Embase (*n* = 135), PubMed (*n* = 102), Scopus (*n* = 107), Cochrane Library (*n* = 4), Web of Science (n = 76), CNKI (n = 177), VIP (n = 201), and Wanfang Data (n = 239). After removal of duplicates, 510 records remained. Following title and abstract screening, 101 reports were assessed in full text, of which 66 were excluded. Ultimately, 35 RCTs (21–55) were included. The study selection process is shown in the PRISMA 2020 flow diagram (Figure 1).

**Figure 1:**
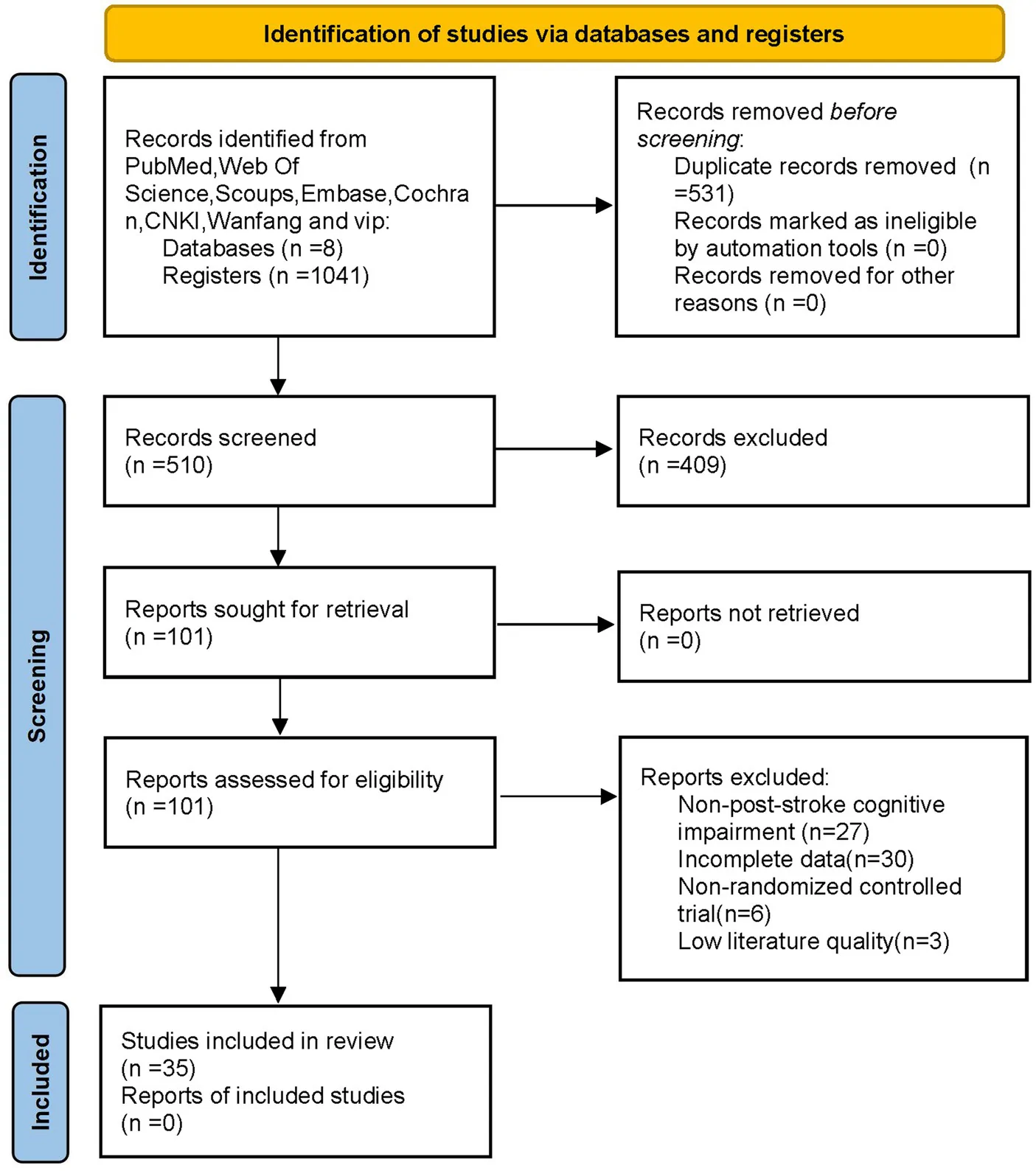
PRISMA 2020 flow diagram of the study selection process.

### 3.2 Characteristics of included studies

A total of 35 RCTs involving 3,036 patients were included. Of these, 1,520 patients (50.1%) were assigned to the HBOT groups and 1,516 (49.9%) to the control groups. Study sample sizes ranged from 12 to 139 participants. The studies were published between 2014 and 2026, with 25 (71.43%) published in 2020 or later. Detailed study characteristics are presented in Table 1.

**Table 1:** Characteristics of the included studies.

Study (Author, Year)	Sample Size		Gender (Male/Female)		Age (years)		Intervention		Hyperbaric Oxygen Parameters			Outcomes
	E group	C group	E group	C group	E group	C group	E group	C group	Pressure (ATA)	Total Interventions	Duration (min)	
Li (2026) (26)	52	52	30/22	28/24	61.54 ± 3.58	61.85 ± 3.70	HBOT+C	Dual-Task Training+Conventional Intervention	2	30	100	MoCA MMSE MBI
Ren (2018) (53)	31	31	17/14	18/13	58.6 ± 6.8	59.2 ± 7.4	HBOT+C	Basic Medication+Conventional Physical Rehabilitation	2	30	110	MoCA MMSE
Zhou (2024) (31)	44	44	24/20	23/21	59.25 ± 6.44	59.22 ± 6.47	HBOT+C	Phased Physical Rehabilitation+Conventional Training	2	20	105	MoCA MMSE NIHSS
Wang (2026) (39)	34	36	22/12	22/14	66.12 ± 13.45	62.43 ± 11.74	HBOT+C	Conventional Treatment+Cognitive Training	1.8	30–42	90	MoCA
Yang (2024) (42)	49	46	27/22	20/26	71.29 ± 5.70	69.70 ± 4.54	HBOT+C	Conventional Drugs+Donepezil	2.2	10	110	MoCA MMSE
Wang (2020) (44)	139	139	78/61	80/59	60.34 ± 3.82	60.25 ± 3.71	HBOT+C	Conventional Pharmacotherapy+Donepezil Hydrochloride	2	20	110	MoCA MMSE BI
Zhang (2014) (49)	50	50	30/20	28/22	66.18 ± 6.89	66.10 ± 6.77	HBOT+C	Conventional Pharmacotherapy	2	20	NR	MoCA
Zhang (2021) (46)	42	42	25/17	22/20	62.05 ± 5.42	62.11 ± 7.56	HBOT+C	Basic Treatment	2	20	NR	MoCA MMSE
Qin (2026) (27)	40	40	NR	NR	NR	NR	HBOT+C	Conventional Drugs+Cognitive Training+Physical Rehabilitation	2	20	NR	MoCA MMSE MBI
Zhang (2026) (29)	51	51	23/28	27/24	63.04 ± 6.21	62.15 ± 5.09	HBOT+C	Basic Pharmacotherapy+Comprehensive Rehabilitation+Donepezil Tablets	2	24	110	MoCA MMSE
ZhaoBo (2025) (22)	30	30	16/14	19/11	64.67 ± 6.58	65.21 ± 6.05	HBOT+C	Basic Pharmacotherapy+Oxiracetam Capsules	2	24	60	MoCA MMSE
Ji (2023) (40)	84	84	42/42	44/40	43.28 ± 8.12	42.85 ± 7.75	HBOT+C	Basic Pharmacotherapy+Butylphthalide Soft Capsules	2.0–2.5	40	120	MoCA MMSE
Wang (2018) (32)	50	50	24/26	28/22	73 ± 9	71 ± 12	HBOT+C	Conventional Treatment+Oxiracetam	2.0216	30	60	MoCA MMSE BI
Zhou (2016) (38)	63	63	32/31	33/30	64.8 ± 9.6	65.8 ± 10.4	HBOT+C	Conventional Pharmacotherapy+Oral Donepezil Hydrochloride	2	30	120	MoCA
Deng (2025) (37)	60	60	21/39	25/35	57.25 ± 3.51	56.35 ± 2.89	HBOT+C	Basic Pharmacotherapy+Tenecteplase+Butylphthalide	2	24	NR	MoCA MMSE NIHSS BI
Guo (2024) (47)	14	12	10/4	7/5	53.86 ± 9.05	51.67 ± 11	HBOT+C	Conventional Pharmacotherapy+Comprehensive Rehabilitation	2	20	100	MoCA MMSE MBI
Wang (2024) (36)	29	30	17/12	19/11	56.43 ± 14.50	58.59 ± 10.15	HBOT+C	Basic Drugs+Comprehensive Rehabilitation+Scalp Acupuncture	2	24	105	MoCA MMSE MBI
Luo (2024) (54)	30	30	20/10	19/11	60.65 ± 4.73	60.58 ± 4.69	HBOT+C	Basic Pharmacotherapy+"Zhi San Zhen”	2	30	105	MoCA MMSE BI
Yang (2020) (48)	29	29	20/9	22/7	58.41 ± 8.99	56.55 ± 8.53	HBOT+C	Conventional Internal Medicine+Rehabilitation+Acupuncture	2	20	115	MoCA MBI
Yang (2019) (30)	30	30	15/15	13/17	52.14 ± 7.56	50.10 ± 6.21	HBOT+C	Conventional Pharmacotherapy+Rehabilitation+Scalp Acupuncture	1.2	20	95	MoCA MMSE
Li (2024) (23)	50	50	32/18	30/20	61.2 ± 2.50	62.5 ± 2.20	HBOT+C	Conventional Pharmacotherapy+Routine Rehab	2	20	115	MoCA NIHSS
Zhan (2019) (51)	30	30	NR	NR	NR	NR	HBOT+C	Conventional Pharmacotherapy+Routine Cognitive-Perceptual Training	2	40	120	MoCA MMSE ADL
Xu (2025) (24)	33	34	18/15	24/10	53.58 ± 5.10	53.61 ± 4.53	HBOT+C	Basic Pharmacotherapy+rTMS	2	12	105	MoCA MMSE
Wang (2021) (45)	46	46	26/20	27/19	64.59 ± 3.24	65.28 ± 3.16	HBOT+C	Early Rehabilitation	2	20	85	MoCA MMSE NIHSS
Liu (2026) (55)	30	27	22/8	15/12	51.9 ± 10.6	53.0 ± 11	HBOT+C	Basic Treatment+Routine Cognitive Rehabilitation	1.8	20	100	MoCA MMSE ADL
Yang (2022) (43)	50	50	26/24	28/22	59 ± 8	60 ± 8	HBOT+C	Cognitive Training	2	14	120	MoCA MMSE NIHSS ADL
Zhong (2016) (50)	30	30	16/14	18/12	59.00 ± 7.83	58 ± 5.26	HBOT+C	Conventional Clinical Drugs+Physical Rehabilitation	2	20	110	MoCA MMSE
Lu (2014) (33)	20	20	15/5	16/4	62.20 ± 7.55	61.72 ± 8.37	HBOT+C	Basic Pharmacotherapy+Routine Rehab	2.2	20	105–110	MoCA BI
DengNa (2025) (35)	40	40	26/14	23/17	49.94 ± 4.01	50.35 ± 3.76	HBOT+C	Cognitive Function Training	2.5	20–28	115	MoCA MMSE MBI NIHSS
Di (2020) (21)	40	40	19/21	18/22	57.8 ± 6.0	58.3 ± 5.6	HBOT+C	Basic Pharmacotherapy+Routine Physical Rehabilitation	2	20	110	MoCA MMSE MBI
Qi (2021) (25)	36	36	NR	NR	58.7 ± 7.9	59.3 ± 8.2	HBOT+C	Conventional Pharmacotherapy+Physical Rehabilitation	2	40	110	MoCA MMSE NIHSS BI
Lv (2020) (34)	46	46	29/19	26/20	46.35 ± 11.40	45.91 ± 10.23	HBOT+C	Conventional Pharmacotherapy+Cognitive Training+Oxiracetam	1.2	20	100	MoCA
Duan (2018) (41)	45	45	23/22	26/19	58.3 ± 6.2	55.8 ± 6.7	HBOT+C	Conventional Pharmacotherapy+Cognitive Training+rTMS	2	20	110	MoCA
RenYun (2018) (52)	27	27	13/14	15/12	58.6 ± 7.8	59.1 ± 7.3	HBOT+C	Basic Drugs+Routine Rehabilitation	2	40	110	MoCA MMSE
Mu (2023) (28)	46	46	27/19	26/20	56.88 ± 5.11	56.96 ± 5.55	HBOT+C	Conventional Pharmacotherapy+Cognitive Training+rTMS	2	20	110	MoCA

E group, HBOT group; C group, control group; ATA, atmospheres absolute; HBOT, Hyperbaric Oxygen Therapy; rTMS, repetitive transcranial magnetic stimulation; MoCA, Montreal Cognitive Assessment; MMSE, Mini-Mental State Examination; BI, Barthel Index; MBI, Modified Barthel Index; NIHSS, National Institutes of Health Stroke Scale; ADL, Activities of Daily Living; NR, not reported.

### 3.3 Risk of bias assessment

The risk of bias in the 35 included RCTs was assessed using the Cochrane RoB 2 tool for the primary outcome, MoCA. As shown in Figure 2, one study (36) was judged to be at low risk of bias in the randomization process domain, while 34 had some concerns. In the deviations from intended interventions domain, one study (48) was judged to be at low risk, one (47) at high risk, and 33 had some concerns. All studies were judged to be at low risk in the domains of missing outcome data and measurement of the outcome, whereas all had some concerns regarding selection of the reported result. Overall, one study (47) was judged to be at high risk of bias and 34 had some concerns; none was judged to be at low risk.

**Figure 2:**
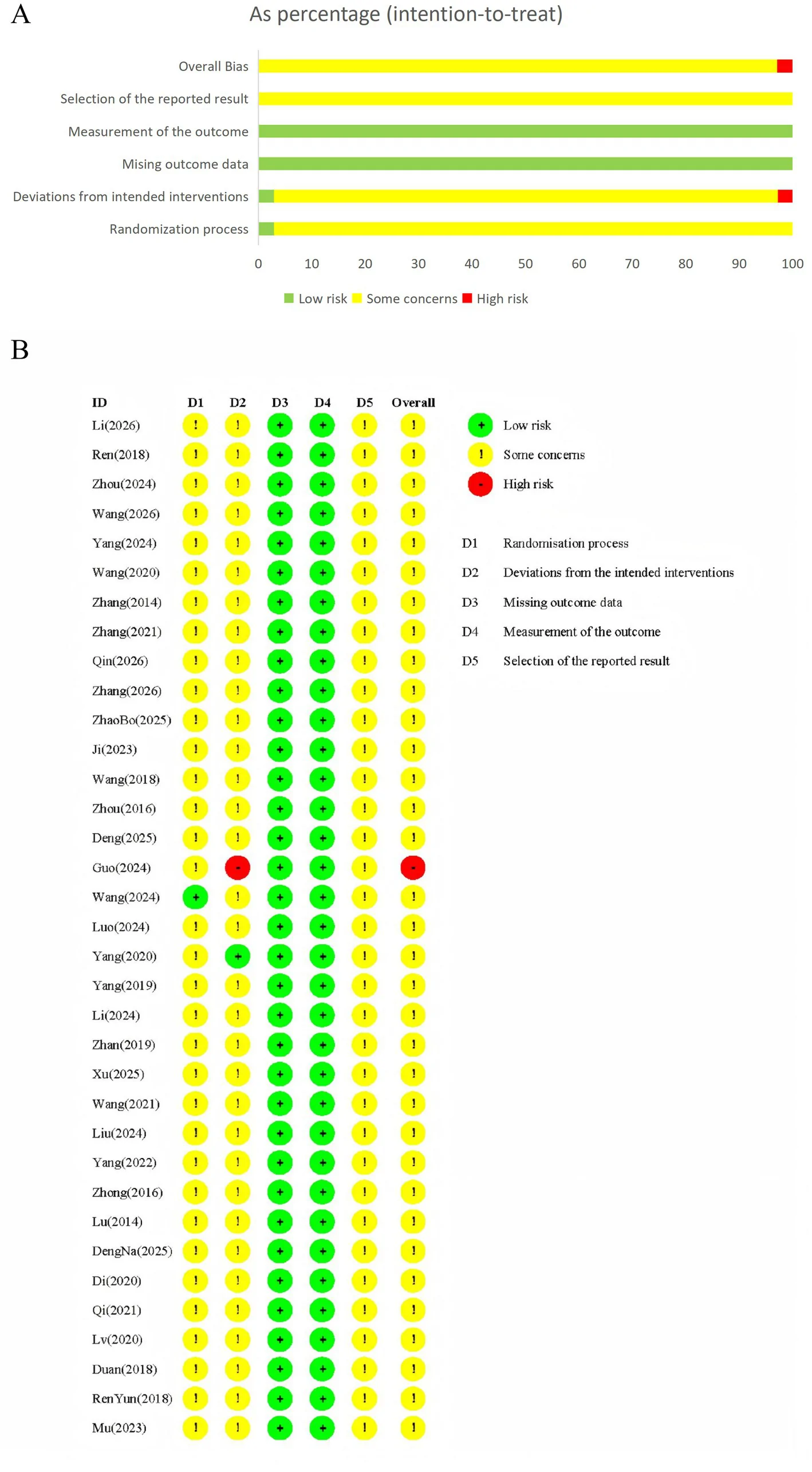
Risk of bias assessment of the included studies. **(A)** Overall risk of bias. **(B)** Risk of bias for individual studies.

### 3.4 Outcome measures

#### 3.4.1 MoCA

All 35 studies reported MoCA scores. HBOT significantly improved MoCA scores compared with conventional treatment alone (MD = 2.90, 95% CI: 2.47–3.33; *p* < 0.00001; *I*^2^ = 84%; Figure 3).

**Figure 3:**
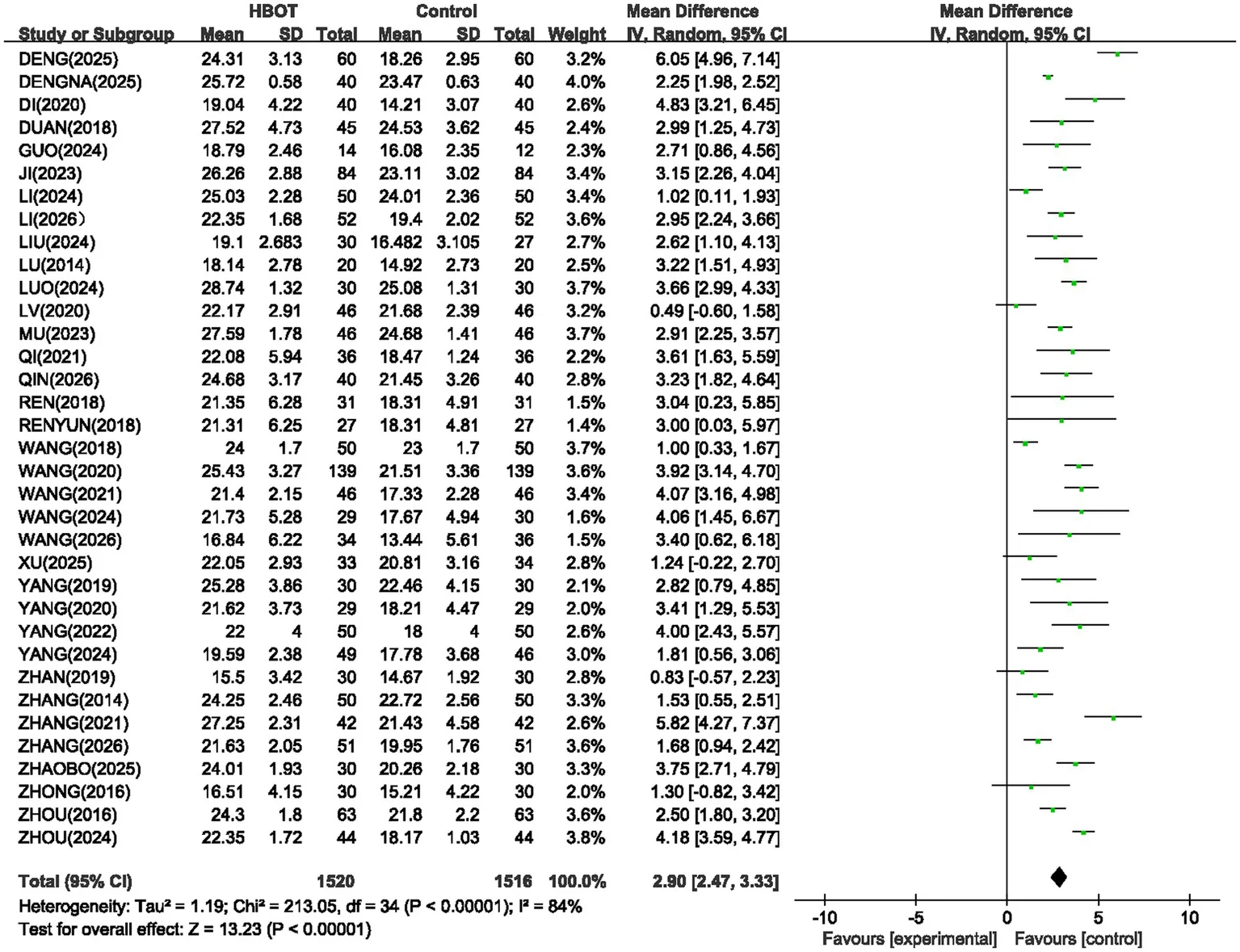
Forest plot of MoCA scores.

#### 3.4.2 MMSE

26 studies (21, 22, 24–27, 29–32, 35–37, 40, 42–47, 50–55) involving 2,268 participants reported MMSE scores (HBOT, n = 1,137; control, n = 1,131). HBOT significantly improved MMSE scores compared with conventional treatment alone (MD = 2.68, 95% CI: 2.20–3.16; *p* < 0.00001; *I*^2^ = 83%; Figure 4).

**Figure 4:**
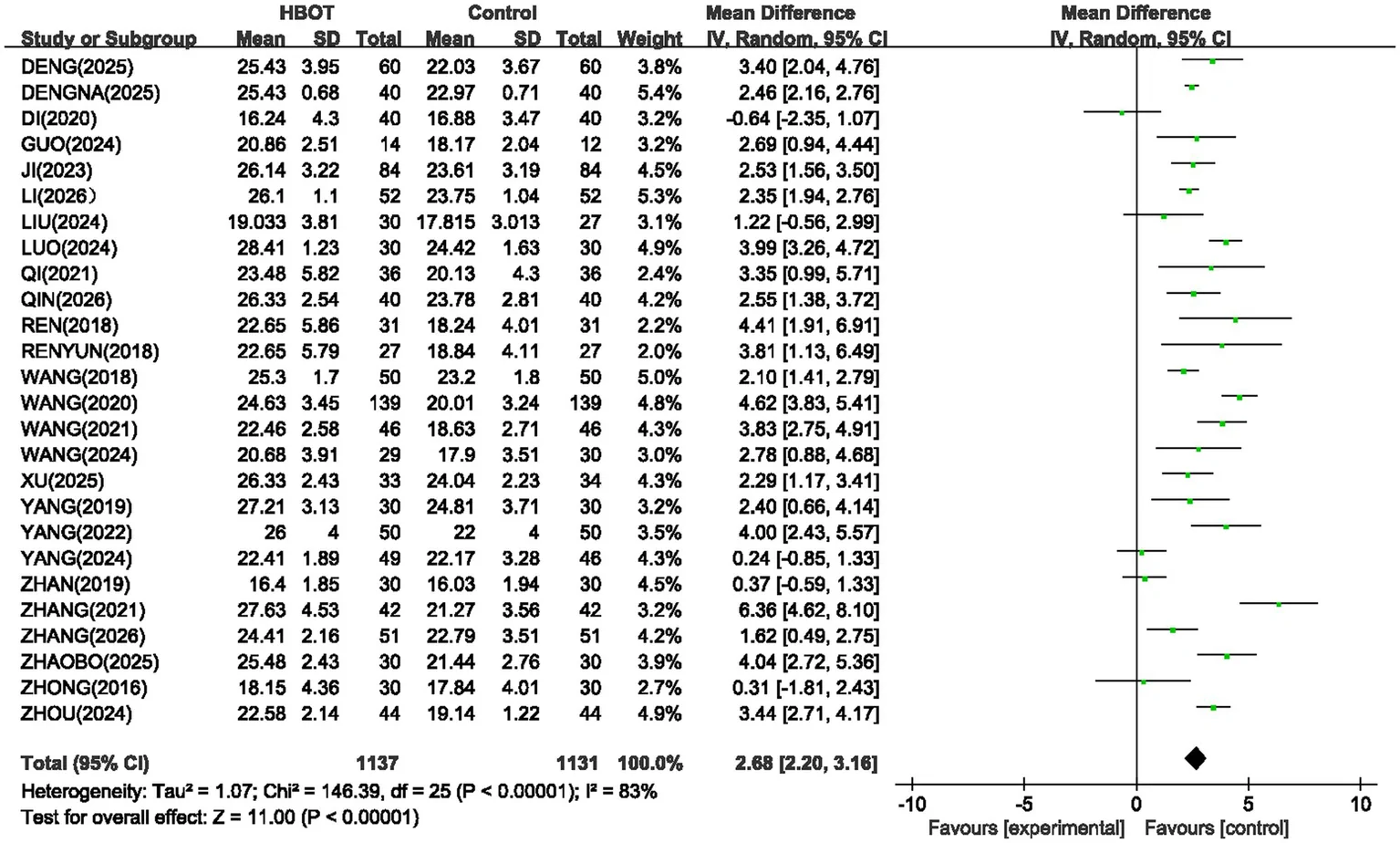
Forest plot of MMSE scores.

#### 3.4.3 NIHSS

7 studies (23, 25, 31, 35, 37, 43, 45) involving 652 participants reported NIHSS scores (HBOT, n = 326; control, n = 326). HBOT significantly reduced NIHSS scores compared with conventional treatment alone (MD = −2.61, 95% CI: −3.34 to −1.89; *p* < 0.00001; *I*^2^ = 93%; Figure 5), indicating improved neurological function.

**Figure 5:**
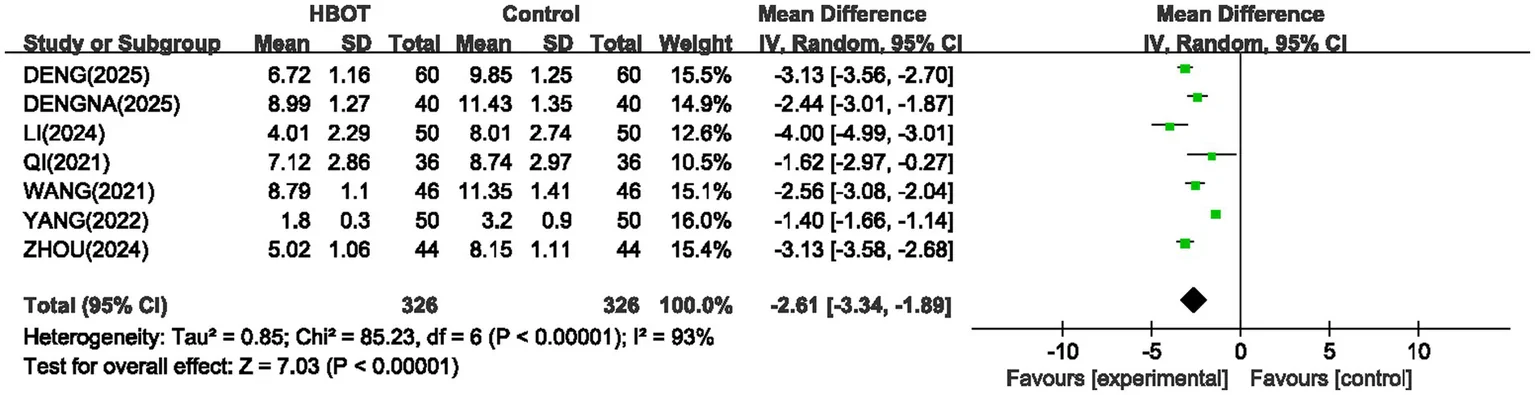
Forest plot of NIHSS scores.

#### 3.4.4 MBI and BI

14 studies assessed activities of daily living, including eight (21, 26, 27, 35, 36, 38, 47, 48) using the MBI and six (25, 32, 33, 37, 44, 54) using the BI. Because the two instruments differ in item composition and scoring, they were analyzed separately. HBOT significantly improved MBI scores (MD = 6.64, 95% CI: 3.76–9.52; *p* < 0.00001; *I*^2^ = 82%; Figure 6) and BI scores (MD = 8.77, 95% CI: 4.72–12.82; *p* < 0.00001; *I*^2^ = 82%; Figure 6).

**Figure 6:**
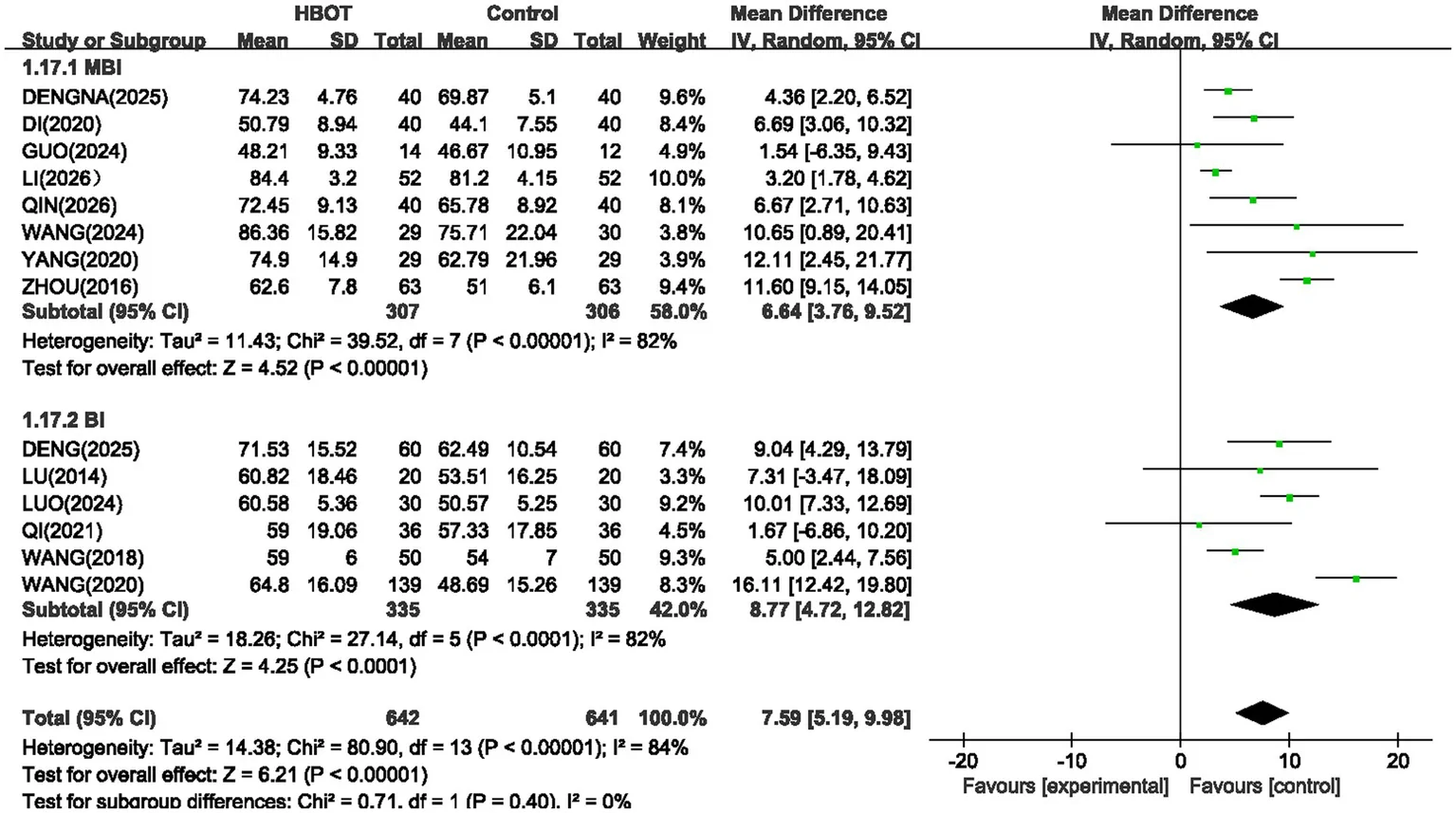
Forest plot of subgroup analyses of MBI and BI scores.

### 3.5 Subgroup analysis

To further explore heterogeneity, subgroup analyses were performed according to the type of conventional treatment received by both the intervention and control groups.

#### 3.5.1 Subgroup analysis of MoCA

Subgroup analyses according to conventional treatment showed significant improvements in MoCA scores with adjunctive HBOT in the medication treatment (MD = 3.23, 95% CI: 2.15–4.31), physical rehabilitation (MD = 3.66, 95% CI: 2.63–4.70), cognitive rehabilitation (MD = 2.59, 95% CI: 1.98–3.20), and acupuncture (MD = 3.59, 95% CI: 3.00–4.18) subgroups (all *p* < 0.00001). Within-subgroup heterogeneity was high in the medication treatment (*I*^2^ = 92%) and cognitive rehabilitation (*I*^2^ = 75%) subgroups, moderate in physical rehabilitation (*I*^2^ = 51%), and absent in acupuncture (*I*^2^ = 0%). The rTMS subgroup contained only three studies and was therefore not included in the formal test for subgroup differences. No statistically significant difference was observed between subgroups (*p* = 0.10; I^2^ = 52.3%) (Figure 7).

**Figure 7:**
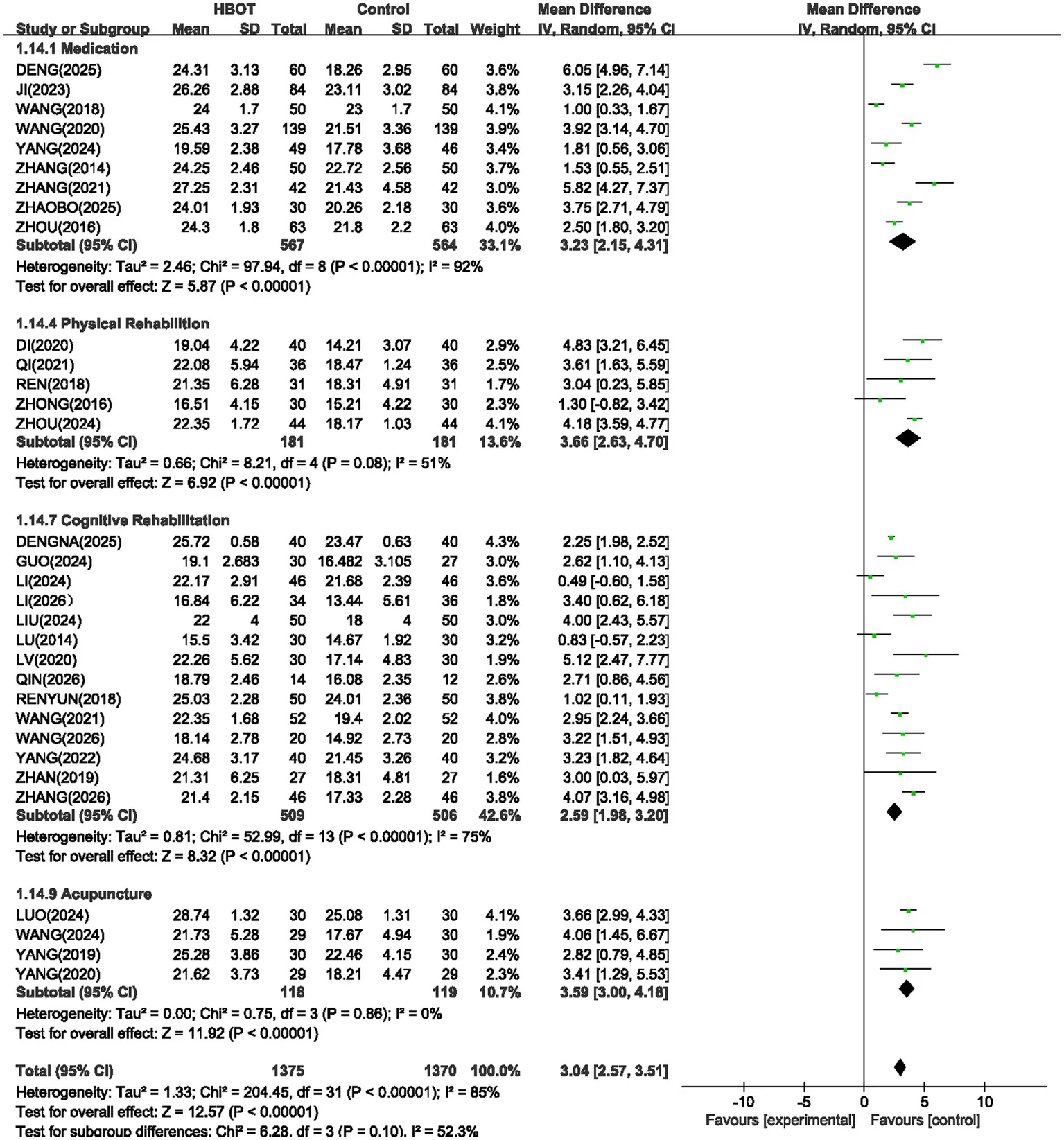
Forest plot of subgroup analysis of MoCA scores by conventional treatment type.

#### 3.5.2 MMSE

Adjunctive HBOT significantly improved MMSE scores in the medication treatment (MD = 3.26, 95% CI: 1.96–4.55; *p* < 0.00001), physical rehabilitation (MD = 2.14, 95% CI: 0.21–4.08; *p* = 0.03), and cognitive rehabilitation (MD = 2.36, 95% CI: 1.81–2.92; *p* < 0.00001) subgroups. Within-subgroup heterogeneity remained high (*I*^2^ = 91, 84, and 73%, respectively), and the test for subgroup differences was not significant (*p* = 0.43, *I*^2^ = 0%; Figure 8).

**Figure 8:**
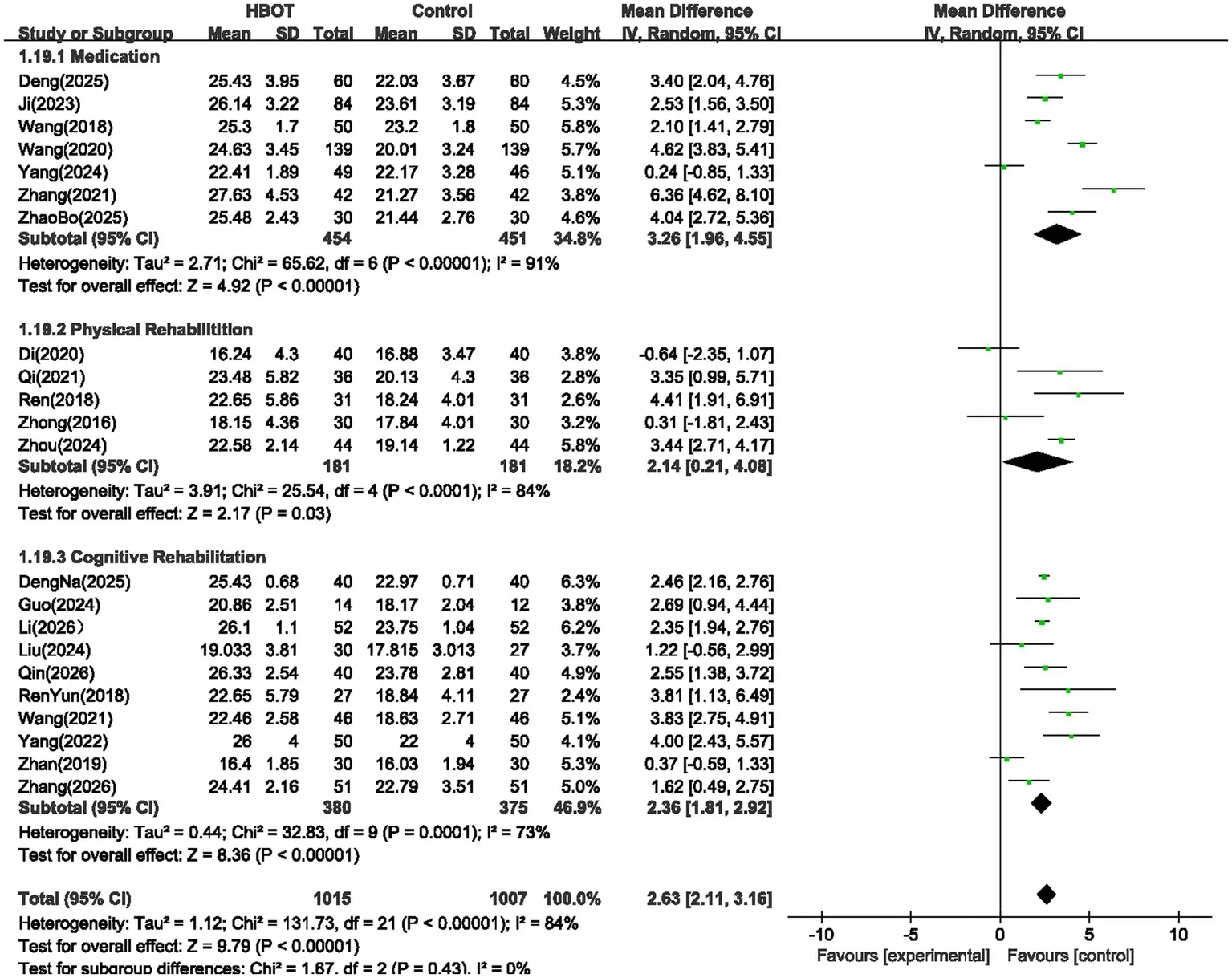
Forest plot of subgroup analysis of MMSE scores by conventional treatment type.

### 3.6 Meta-regression

Univariable random-effects meta-regression examined the associations of total HBOT sessions, baseline MoCA score, and HBOT treatment pressure with treatment effects. No significant associations were observed for treatment sessions (*β* = −0.0042, 95% CI: −0.0636-0.0552; *p* = 0.8892) or treatment pressure (*β* = −2.2548, 95% CI: −20.2214-15.7118; *p* = 0.8057). Baseline MoCA score showed a potential negative association with the treatment effect, with higher baseline MoCA scores tending to be associated with smaller treatment effects; however, this association did not reach statistical significance (*β* = −0.1411, 95% CI:-0.2903–0.0081; *p* = 0.0637; *R*^2^ = 15.42%). This association should be interpreted cautiously given the study-level nature of meta-regression.

### 3.7 Sensitivity analysis and small-study effects

Leave-one-out sensitivity analyses showed consistent effect directions across all outcomes. In the MoCA physical rehabilitation subgroup, removal of one study reduced *I*^2^ from 51 to 0% without changing the effect direction.

Funnel plots and Egger’s tests showed no significant small-study effects for MoCA (*p* = 0.2364; Figure 9A) or MMSE (*p* = 0.7109; Figure 9B). Funnel plots and Egger’s tests were not performed for NIHSS, MBI, or BI because fewer than 10 studies were available.

**Figure 9:**
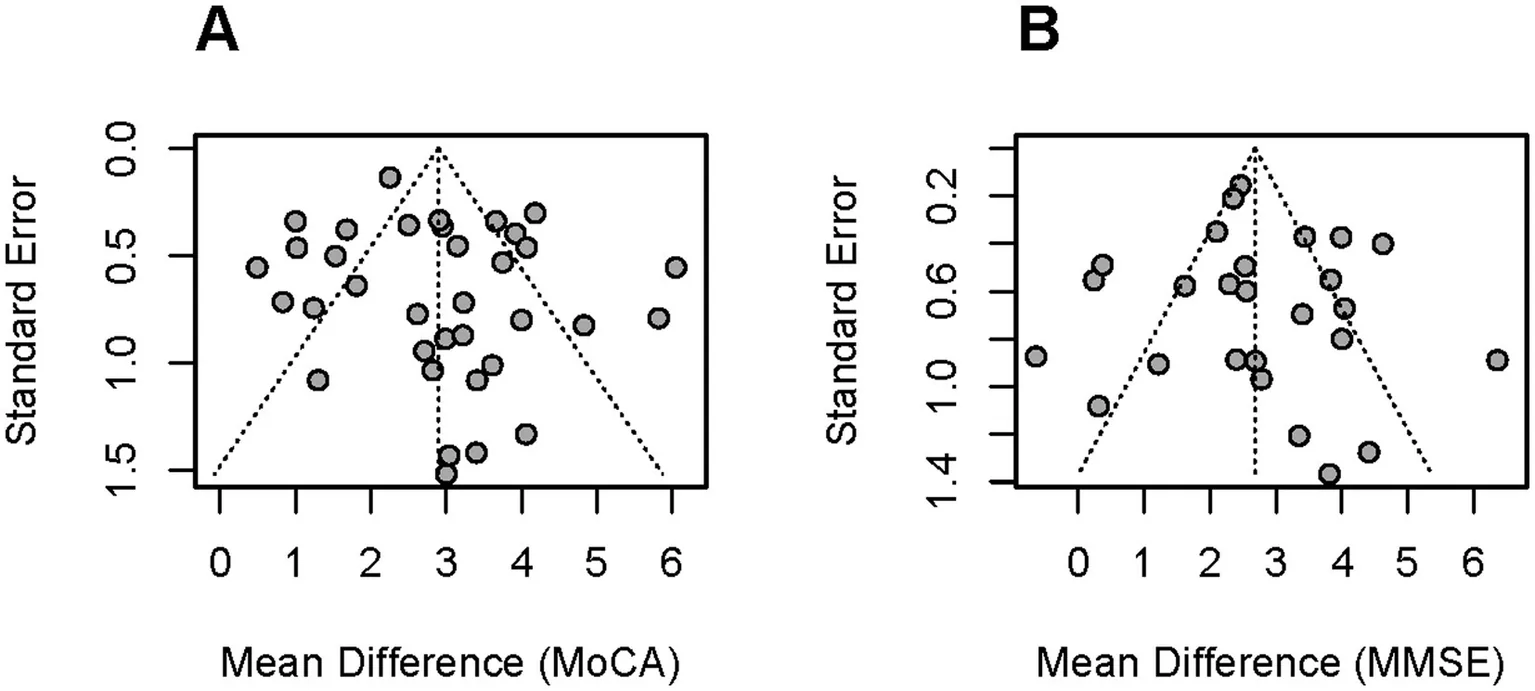
Funnel plots for publication bias assessment. **(A)** MoCA; **(B)** MMSE.

### 3.8 GRADE assessment of the certainty of evidence

The certainty of evidence for each outcome was assessed using the GRADEpro GDT tool. The certainty of evidence was low for MoCA, MMSE, and MBI and very low for NIHSS and BI. Downgrading was primarily due to risk of bias, inconsistency, and imprecision, with no serious concerns regarding indirectness. Detailed GRADE assessments are presented in Table 2.

**Table 2:** GRADE assessment of the certainty of evidence for each outcome.

Certainty assessment							Summary of findings				
Participants (studies) Follow-up	Risk of bias	Inconsistency	Indirectness	Imprecision	Publication bias	Overall certainty of evidence	Study event rates (%)		Relative effect (95% CI)	Anticipated absolute effects	
							With Conventional treatment without HBOT	With Hyperbaric oxygen therapy (HBOT)		Risk with Conventional treatment without HBOT	Risk difference with Hyperbaric oxygen therapy (HBOT)
MoCA (assessed with: Montreal Cognitive Assessment; Scale from: 0 to 30)											
3,036 (35 RCTs)	Serious^a^	Serious^b^	Not serious	Not serious	None	⨁⨁◯◯ Low^a,b^	1,516	1,520	-	1,516	MD 2.9 higher (2.47 higher to 3.33 higher)
MMSE (assessed with: Mini–mental state examination; Scale from: 0 to 30)											
2,268 (26 RCTs)	Serious^a^	Serious^b^	Not serious	Not serious	None	⨁⨁◯◯ Low^a,b^	1,131	1,137	-	1,131	MD 2.68 higher (2.2 higher to 3.16 higher)
NIHSS (assessed with: National Institutes of Health Stroke Scale; Scale from: 0 to 42)											
652 (7 RCTs)	Serious^a^	serious^b^	not serious	serious^c^	none	⨁◯◯◯ Very low^a,b,c^	326	326	-	326	MD 2.61 lower (3.34 lower to 1.89 lower)
MBI (assessed with: Modified Barthel Index; Scale from: 0 to 100)											
613 (8 RCTs)	Serious^a^	Serious^b^	Not serious	Not serious	None	⨁⨁◯◯ Low^a,b^	306	307	-	306	MD 6.64 higher (3.76 higher to 9.52 higher)
BI (assessed with: Barthel Index; Scale from: 0 to 100)											
670 (6 RCTs)	Serious^a^	Serious^b^	Not serious	Serious^c^	None	⨁◯◯◯ Very low^a,b,c^	335	335	-	335	MD 8.77 higher (4.72 higher to 12.82 higher)

CI, confidence interval; MD, mean difference. a, downgraded one level for serious risk of bias; b, downgraded one level for serious inconsistency; c, downgraded one level for serious imprecision. GRADE certainty levels: ⊕⊕⊕⊕, high; ⊕⊕⊕◯, moderate; ⊕⊕◯◯, low; ⊕◯◯◯, very low.

### 3.9 Adverse events

Six of the 35 included studies reported adverse events. In the HBOT groups, reported events mainly included gastrointestinal discomfort, nausea, insomnia, ear fullness, and ear pain. Control-group events mainly included acupuncture-related bleeding and vasovagal reactions. One study reported edema and dyspepsia in the HBOT group and myocardial infarction and pulmonary infection in the control group. Given the limited reporting, the available data were insufficient to comprehensively assess the safety of HBOT (56).

## 4 Discussion

### 4.1 Main findings

This systematic review included 35 randomized controlled trials involving 3,036 patients with PSCI and evaluated the effects of adjunctive HBOT on cognitive, neurological, and functional outcomes. Overall, HBOT added to conventional treatment was associated with improvements in MoCA and MMSE scores and was associated with favorable effects on NIHSS, MBI, and BI. These findings suggest that HBOT may provide additional benefits for cognitive and functional recovery in patients with PSCI.

The consistent improvements in MoCA and MMSE are broadly consistent with previous evidence suggesting potential cognitive benefits of HBOT in neurological disorders (57). Notably, the pooled improvement in MoCA (MD = 2.90 points) exceeded previously reported MCID estimates of 1.0–2.15 points in stroke populations, suggesting potential clinical relevance (58, 59). However, previous studies have reported heterogeneous effects across diseases, cognitive domains, and treatment protocols. The present review specifically focused on PSCI and included only RCTs, providing more direct evidence regarding the potential adjunctive role of HBOT in this population.

The favorable effects on NIHSS, MBI, and BI suggest that the potential benefits of HBOT may extend beyond cognition to neurological function and activities of daily living. Differences from previous HBOT studies in acute ischemic stroke may reflect differences in patient populations, treatment objectives, and the use of HBOT as an adjunct to rehabilitation. Therefore, these findings should not be extrapolated to HBOT as a treatment for acute stroke reperfusion.

### 4.2 Potential mechanisms

HBOT may facilitate post-stroke neurological recovery by increasing cerebral oxygen availability and modulating the post-ischemic microenvironment. Potential mechanisms include modulation of oxidative stress, inflammation, apoptosis, mitochondrial function, and neurovascular remodeling, which may contribute to recovery of injured neural tissue (57).

HBOT may also influence neuroplasticity and functional brain-network reorganization. One RCT included in this review reported that HBOT combined with cognitive training improved MMSE and BI scores and enhanced functional connectivity involving the left dorsolateral prefrontal cortex and Broca’s area; changes in functional connectivity were correlated with MMSE improvement (55). These findings suggest a possible association between functional connectivity changes and cognitive improvement following adjunctive HBOT, although the mechanistic significance of this association remains uncertain.

Importantly, HBOT should be considered an adjunctive rather than a replacement therapy. Most included studies compared HBOT plus conventional treatment with conventional treatment alone, and therefore primarily evaluated the incremental effects of adding HBOT to conventional treatment.

### 4.3 Heterogeneity

Substantial heterogeneity was observed across the major outcomes. Subgroup analyses suggested that conventional treatment type might contribute to the heterogeneity in MoCA effects; however, the difference between subgroups was not statistically significant. Substantial residual heterogeneity remained in several subgroups, indicating that treatment type alone could not fully account for the observed variability. In contrast, no significant subgroup differences were observed for MMSE.

Meta-regression identified baseline MoCA score as a potential study-level moderator, with higher baseline MoCA scores tending to be associated with smaller treatment effects, explaining approximately 15.42% of the between-study variance. No significant associations were observed for the number of HBOT sessions or treatment pressure. These findings should be interpreted cautiously because meta-regression is based on study-level rather than individual-level data and does not establish effect modification at the patient level.

The results suggest that baseline cognitive status may contribute to variation in treatment effects, but the current evidence is insufficient to define an optimal target population. Future trials should stratify patients according to baseline cognitive severity, and individual participant data meta-analysis may help clarify potential effect modifiers.

### 4.4 Robustness and certainty of evidence

Sensitivity analyses showed consistent effect directions across outcomes, suggesting that the findings were not driven by individual studies. However, substantial heterogeneity persisted across outcomes and within most subgroups. Meta-regression identified baseline MoCA as a potential source of heterogeneity, but it explained only 15.42% of the between-study variance. Thus, most heterogeneity remained unexplained, and the pooled estimates should be interpreted cautiously (18, 60). The certainty of evidence was low for MoCA, MMSE, and MBI and very low for NIHSS and BI, further limiting confidence in the estimated effects.

Only six studies reported adverse events, limiting the assessment of HBOT-related risks. The absence of reported adverse events in other studies does not establish their absence (56). Previous evidence has also identified HBOT-related adverse effects, particularly ear discomfort (61). Furthermore, although funnel plots and Egger’s tests did not detect significant small-study effects for MoCA or MMSE, publication bias cannot be excluded because these methods have limited power. The exclusively Chinese evidence base may also limit generalizability.

### 4.5 Clinical implications

The findings of this review suggest that adjunctive HBOT may have potential benefits for cognitive and functional recovery in patients with PSCI. However, the clinical implications of these findings should be interpreted cautiously. Most included studies compared HBOT plus conventional treatment with conventional treatment alone; therefore, the observed effects represent the potential incremental benefits of adding HBOT to existing care rather than its effects as a standalone treatment.

The applicability of these findings to international clinical practice remains uncertain, as all included studies were conducted in China. Differences in patient characteristics, diagnostic criteria for PSCI, HBOT protocols, and concomitant rehabilitation practices across healthcare settings may influence treatment responses and the interpretation of pooled effect estimates. In particular, differences in cognitive assessment thresholds may affect the characteristics of enrolled patients, while variation in background treatments may influence the incremental benefits associated with HBOT. These potential differences were not directly evaluated in this review and should not be interpreted as established differences between China and other countries.

Furthermore, substantial between-study heterogeneity and low-to-very-low certainty of evidence limit confidence in the pooled estimates. Although the findings suggest potential benefits of adjunctive HBOT, they do not establish its effectiveness across different populations or healthcare systems. The current evidence therefore does not support the routine use of HBOT in all patients with PSCI. Future well-designed, multicenter randomized controlled trials involving diverse populations and healthcare settings, with standardized diagnostic criteria, clearly reported HBOT protocols, and systematic safety monitoring, are needed to establish its clinical applicability.

### 4.6 Limitations

Several limitations should be acknowledged. First, methodological limitations, particularly difficulties in blinding, may have affected the reliability of the findings. Second, substantial clinical heterogeneity remained largely unexplained despite subgroup analyses and meta-regression. Third, incomplete reporting of HBOT parameters and limited long-term follow-up restricted the assessment of dose–response relationships and the durability of treatment effects. Fourth, only six of the 35 included studies reported adverse events, limiting safety assessment and precluding the exclusion of unreported rare or serious events (61). Fifth, all included studies were conducted in China, limiting the generalizability of the findings. Although funnel plots and Egger’s tests detected no significant small-study effects for MoCA or MMSE, publication bias cannot be excluded because these methods have limited power.

## 5 Conclusion

Adjunctive HBOT may provide additional cognitive and functional benefits in patients with PSCI. However, substantial between-study heterogeneity, low-to-very-low certainty of evidence, and the exclusively Chinese evidence base jointly warrant cautious interpretation and limit the applicability of the findings to international clinical practice. Large, well-designed randomized controlled trials involving diverse populations, with long-term follow-up and systematic safety monitoring, are needed to confirm the effects and safety of adjunctive HBOT in patients with PSCI.

## Data Availability

The original contributions presented in the study are included in the article/Supplementary material, further inquiries can be directed to the corresponding author.
