# Supplementary material for: Adjunctive hyperbaric oxygen therapy for post-stroke cognitive impairment: a systematic review and meta-analysis of randomized controlled trials
Source: Front Neurol. 2026 Oct 13;17:1982226. doi: 10.3389/fneur.2026.1982226 (PMC13710107; doi:10.3389/fneur.2026.1982226)
Supplement: Supplementary file 1 [file Data_Sheet_1.pdf]

Embase：
('stroke'/exp OR 'stroke*' OR 'poststroke' OR 'cerebral infarction' OR 'cerebral hemorrhage' OR 'cerebrovascular accident' OR 'cva' OR 'apoplexy') AND ('cognition disorders'/exp OR 'cognit*' OR 'memory' OR 'executive function' OR 'attention' OR 'psci' OR 'moca' OR 'mmse') AND ('hyperbaric oxygenation'/exp OR 'hbot' OR 'hbo' OR 'hyperbaric oxygen') AND ('article'/it OR 'clinical trial'/it)

PUBMED:
("Stroke"[Mesh] OR stroke* OR poststroke OR cerebral infarction OR cerebral hemorrhage OR cerebrovascular accident OR CVA OR Apoplexy) AND ("Cognition Disorders"[Mesh] OR cognit* OR memory OR executive function OR attention OR PSCI OR MoCA OR MMSE) AND ("Hyperbaric Oxygenation"[Mesh] OR hyperbaric oxygen OR HBOT OR HBO)

Scoup:
(“stroke*” OR “poststroke” OR “cerebrovascular accident" OR "cerebral infarction" OR "cerebral hemorrhage" OR “CVA” OR “Apoplexy”) AND (“Cognition Disorders” OR “cognit*” OR “memory” OR "executive function" OR “PSCI” OR “MoCA” OR “MMSE”) AND ("hyperbaric oxygen" OR "hyperbaric oxygen therapy" OR “HBOT” OR “HBO”)

Cochrane:

#1	MeSH descriptor: [Stroke] explode all trees	梅什	19822
#2	MeSH descriptor: [Cerebral Infarction] explode all trees	梅什	1723
#3	MeSH descriptor: [Cerebral Hemorrhage] explode all trees	梅什	1706
#4	poststroke	界限	8792
#5	MeSH descriptor: [Cognition Disorders] explode all trees	梅什	9819
#6	MeSH descriptor: [Cognition] explode all trees	梅什	17685
#7	MeSH descriptor: [Memory] explode all trees	梅什	10339
#8	MeSH descriptor: [Executive Function] explode all trees	梅什	2159
#9	Psci	界限	197
#10	moca	界限	4033
#11	MeSH descriptor: [Mental Status and Dementia Tests] explode all trees	梅什	890
#12	MeSH descriptor: [Hyperbaric Oxygenation] explode all trees	梅什	642
#13	HBOT	界限	429
#14	HBO	界限	481
#15	#1 OR #2 OR #3 OR #4	界限	26495
#16	#5 OR #6 OR #7 OR #8 OR #9 OR #10 OR #11	界限	37181
#17	#12 OR #13 OR #14	界限	1187
#18	#15 AND #16 AND #17	界限	5

Web of science:
(“stroke*” OR “poststroke” OR “cerebrovascular accident" OR "cerebral infarction" OR "cerebral hemorrhage" OR “CVA” OR “Apoplexy”) AND (“Cognition Disorders” OR “cognit*” OR “memory” OR "executive function" OR “PSCI” OR “MoCA” OR “MMSE”) AND ("hyperbaric oxygen" OR "hyperbaric oxygen therapy" OR “HBOT” OR “HBO”)

知网检索表达式：
(TI = '高压氧' OR KY = '高压氧' OR TI = '高压氧治疗' OR KY = '高压氧治疗') AND (TI= '认知障碍' OR TI = '认知功能' OR TI = '认知' OR TI = '记忆' OR TI = '

执行功能' OR KY = '认知障碍' OR KY = '认知功能' OR KY = '认知' OR KY = '记忆' OR KY = '执行功能') AND (TI= '脑卒中' OR TI = '卒中' OR TI = '脑梗死' OR TI = '脑出血' OR TI = '中风' OR TI = '脑栓塞' OR TI = '脑梗塞' OR TI = '脑缺血 'OR TI = '脑溢血'OR TI = '脑血管意外' OR KY = '脑卒中' OR KY = '卒中' OR KY = '脑梗死' OR KY = '脑出血' OR KY = '中风' OR KY = '脑栓塞' OR KY = '脑梗塞' OR KY = '脑缺血' OR KY = '脑溢血' OR KY = '脑血管意外')

万方检索表达式：

(题名或关键词:(“高压氧”) or 题名或关键词:(“高压氧治疗”)) and (题名或关键词:(“认知障碍”) or 题名或关键词:(“认知功能”) or 题名或关键词:(“认知”) or 题名或关键词:(“记忆”) or 题名或关键词:(“执行功能”)) and (题名或关键词:(“脑卒中”) or 题名或关键词:(“卒中”) or 题名或关键词:(“脑梗死”) or 题名或关键词:(“脑出血”) or 题名或关键词:(“中风”) or 题名或关键词:(“脑栓塞”) or 题名或关键词:(“脑梗塞”) or 题名或关键词:(“脑缺血”) or 题名或关键词:(“脑溢血”) or 题名或关键词:(“脑血管意外”))

维普检索表达式：

(M=高压氧 OR M=高压氧治疗) AND (M=认知障碍 OR M=认知功能 OR M=认知 OR M=记忆 OR M=执行功能) AND (M=脑卒中 OR M=卒中 OR M=脑梗死 OR M=脑出血 OR M=中风 OR M=脑栓塞 OR M=脑梗塞 OR M=脑缺血 OR M=脑溢血 OR M=脑血管意外)
